# Two Autochthonous Cases of Anaplasmosis, Washington, USA, 2022–2023

**DOI:** 10.3201/eid3111.250379

**Published:** 2025-11

**Authors:** Hannah Schnitzler, Mary Chan, Jeni Nybo, Kelley Palmer-McGee, Zachary Doobovsky, Ian Tracy, Siu-Kei Chow, Roumen B. Iordanov, Eugene H. Lee, Julianna R. Van Enk, Elizabeth A. Dykstra, Beth A. Lipton, Hanna N. Oltean

**Affiliations:** Washington State Department of Health, Olympia, Washington, USA (H. Schnitzler, M. Chan, E.A. Dykstra, B.A. Lipton, H.N. Oltean); Tacoma-Pierce County Health Department, Tacoma, Washington, USA (J. Nybo); Whatcom County Health and Community Services, Bellingham, Washington, USA (K. Palmer-McGee, Z. Doobovsky); Mason County Public Health and Human Services, Shelton, Washington, USA (I. Tracy); MultiCare Health System, Tacoma (S.-K. Chow, R.B. Iordanov, E.H. Lee, J.R. Van Enk)

**Keywords:** Anaplasmosis, *Anaplasma phagocytophilum*, vector-borne infections, bacteria, tickborne diseases, tick, tick bite, *Ixodes*, *Ixodes pacificus*, Washington State

## Abstract

We describe 2 cases of autochthonous human anaplasmosis in Washington, USA, where anaplasmosis has been rarely reported. Clinicians should consider anaplasmosis in the differential diagnosis for patients with compatible clinical symptoms after tick bite or time spent outdoors in an area where *Ixodes pacificus* ticks are present.

Anaplasmosis is a tickborne disease caused by *Anaplasma phagocytophilum*, a bacterium spread by some *Ixodes* spp. ticks (*1*). *I. pacificus*, the western black-legged tick, is the primary vector for *A. phagocytophilum* on the West Coast of the United States (*2*). Most patients with anaplasmosis experience moderate illness, including fever, malaise, headache, myalgia, nausea, vomiting, or diarrhea (*3*–*6*). The disease can progress to severe illness, with 31% of reported case-patients hospitalized, and rarely to death; the case-fatality rate is 0.3% in the United States (*7*). The risk for severe illness increases with advanced patient age, immunosuppression, and delayed diagnosis and treatment (*6*,*7*). Laboratory testing often shows transaminitis and cytopenias, including anemia, thrombocytopenia, and leukopenia (*3*–*6*). Peripheral blood smear tests may show morulae in granulocytes during acute illness, but that test is not a sensitive method for diagnosis (*4*,*8*). Definitive diagnosis relies on molecular testing, immunohistochemistry, or culture. Serologic testing is less specific (*4*).

The seasonality of human anaplasmosis cases in the United States coincides with vector activity. Nymphal *Ixodes* spp. ticks are active during March through early July; most anaplasmosis cases in the United States occur during May−August. A smaller peak in cases takes place during October−November, when adult *Ixodes* ticks are active (*9*).

In Washington, USA, the range of *I. pacificus* ticks encompasses western Washington and the eastern slopes of the Cascade mountains (*10*). Tick surveillance conducted by the Washington State Department of Health during 2011−2017 identified *A. phagocytophilum* in *I. pacificus* and *I. spinipalpis* ticks (*2*). Despite detection of *A. phagocytophilum* in ticks in the state, reports of autochthonous cases are rare but have been documented in canines with no recent travel outside of western Washington (*11*). We describe 2 reported human cases of autochthonous anaplasmosis in Washington.

## The Case-Patients

In July 2022, an 81-year-old man (case-patient 1) visited an urgent care center with symptoms of fever (starting that day), shortness of breath, and dizziness. His medical history was notable for paroxysmal atrial fibrillation, congestive heart failure with dyspnea on exertion, pulmonary embolism, hypertension, dyslipidemia, and stroke. Bloodwork revealed thrombocytopenia (78,000 platelets/µL; reference range 140,000–400,000 platelets/µL), elevated aspartate aminotransferase (58 U/L; reference 10–35 U/L), and acute kidney injury with elevated blood urea nitrogen (32 mg/dL; reference 7–25 mg/dL) and creatinine (1.51 mg/dL; reference 0.70–1.22 mg/dL). Treating physicians discharged the patient with instructions to return for care if his symptoms worsened.

The patient sought treatment at an emergency department 2 days later for worsening shortness of breath, fatigue, weakness, and fever reaching 103°F. His platelet count had worsened to 44,000 platelets/µL, and his aspartate aminotransferase level was 86 U/L. A computed tomography angiogram identified mild cardiomegaly and trace bilateral pleural effusion but no pulmonary embolism. Computed tomography of the abdomen and pelvis revealed unremarkable results, and results of a respiratory virus panel (Biofire Respiratory 2.1 Panel, https://www.biofiredx.com) were negative. The patient received a dose of ceftriaxone and was admitted to the hospital. Consultation with infectious disease specialists prompted blood sample collection for tickborne disease testing, and physicians initiated empiric doxycycline 3 days after admission. The patient showed improvement in platelet count and liver function on that day and was discharged. Five days after discharge, the patient’s blood sample results returned positive for *A. phagocytophilum* by qualitative real-time PCR conducted at a commercial laboratory, and physicians prescribed a continued 10-day course of doxycycline. His symptoms resolved ≈1 month after discharge.

The commercial laboratory reported the patient’s PCR results to the local health jurisdiction, who forwarded the sample to the Centers for Disease Control and Prevention (Atlanta, GA, USA), where real-time PCR and sequence analysis confirmed *A. phagocytophilum.* Upon interview, the patient reported no travel outside of Washington state and no tick detections or tick bites during the exposure period (5−21 days before symptom onset). Three weeks before symptom onset, the patient did visit Mason County, Washington, for several days and performed yard work in an area where a neighbor recently reported a tick bite. Public health officials presumed that location to be the patient’s likely exposure location. Environmental investigation did not begin until April 2023, because nymphal ticks are not likely to be active in August; however, limited drag sampling in April revealed no ticks for collection.

In June 2023, a woman (case-patient 2) residing in Washington began experiencing fever, back pain, neck stiffness, and headache. The woman had a history of psoriasis and hypothyroidism, managed with levothyroxine and a topical corticosteroid. Four days after symptom onset, she visited an urgent care center, where peripheral blood smear testing showed evidence of circulating, unidentified atypical cells and neutropenia, suggesting an infectious disease process or leukemia. Attending physicians discharged the patient with instructions to seek care if symptoms worsened. The woman visited an emergency department 2 days later, reporting fever, severe headache, back pain, and neck stiffness. Physicians admitted her to the hospital, where bloodwork revealed leukopenia (leukocytes 2,540/µL; reference range 4,000–12,000/µL), thrombocytopenia (platelets 96,000/µL; referenced 150,000–450,000/µL), anemia (erythrocytes 3.5 million/µL; reference 4–5.5 million/µL), and transaminitis (alanine transaminase 691 IU/L, reference 6–60 IU/L; aspartate aminotransferase 786 IU/L, reference 5–40 IU/L; alkaline phosphatase 435 IU/L, reference 28–126 IU/L). Computed tomography showed hepatosplenomegaly but no other abnormalities, and additional serologic testing showed negative results for cytomegalovirus, hepatitis (A, B, and C), HIV, and *Toxoplasma.* A respiratory virus panel (VERIGENE Respiratory Pathogens *Flex* Test, https://us.diasorin.com) and 2 sets of blood cultures revealed negative results. The patient tested positive for group A *Streptococcus* on throat culture and had low-level viremia with Epstein-Barr virus, although antibody testing was negative. An infectious disease specialist consulted on the day of admission recommended an extensive work-up for viral and tickborne diseases, and attending physicians initiated a course of cefepime and doxycycline the next day. A blood sample collected the day after admission tested positive for *A. phagocytophilum* via real-time PCR on day 6 of hospitalization, and physicians prescribed a continuing regimen of doxycycline for 10 days. Thrombocytopenia and leukopenia resolved 4 days after starting doxycycline, and physicians discharged the patient 10 days after admission, noting gradual improvement in symptoms.

The hospital laboratory reported the real-time PCR results for this patient to officials at the local health jurisdiction, who interviewed the patient. The patient reported hiking in multiple parks in Pierce County, but no travel outside of Washington during the exposure period. No environmental investigation took place because the patient did not report a tick bite and researchers could identify no single exposure location.

## Conclusion

Washington is a low incidence region for tickborne diseases (*2*), posing a challenge for public health surveillance, prevention communication, and provider education. We charted the counties in Washington with documented *I. pacificus* tick presence and the suspected counties of exposure for the 2 human cases of anaplasmosis (Figure).

**Figure:**
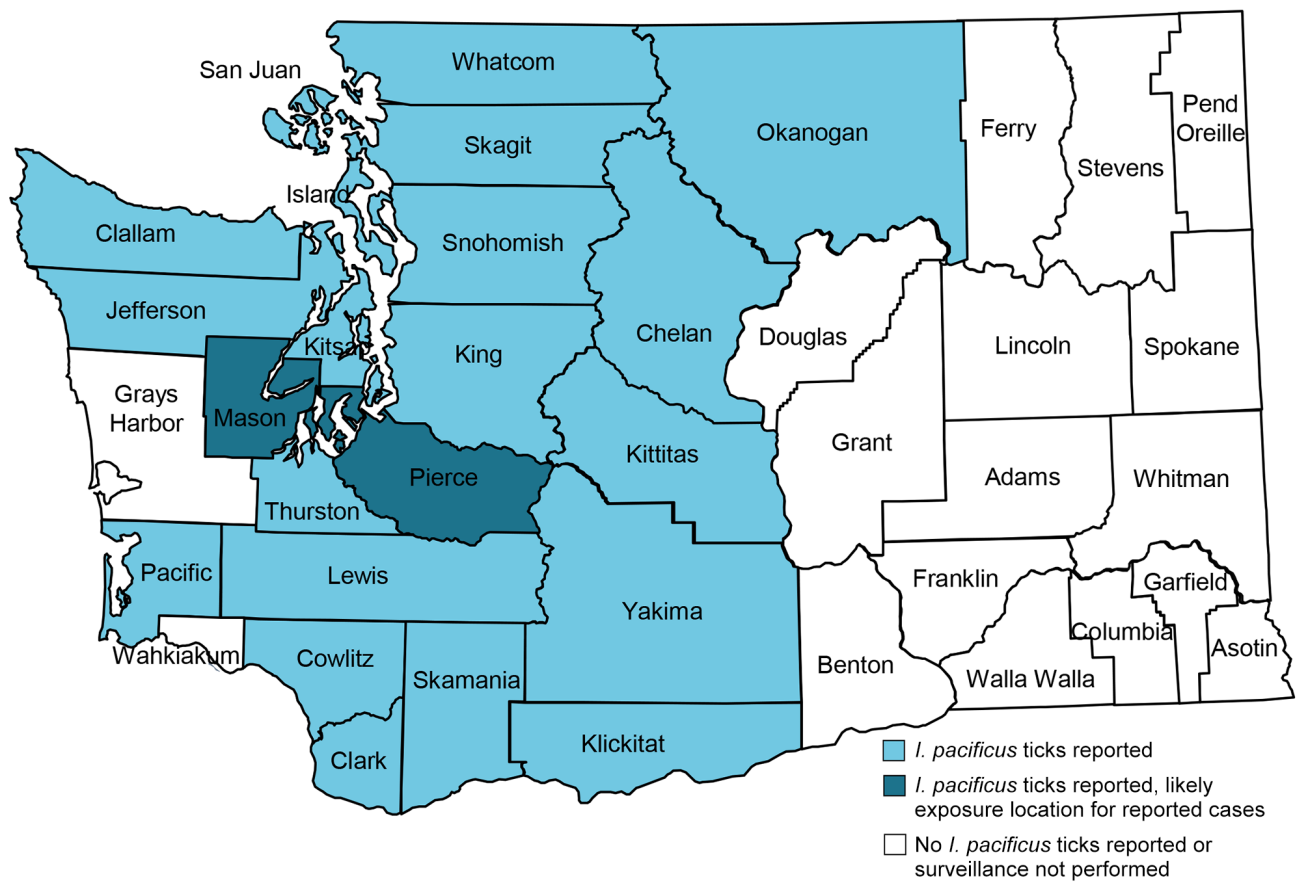
Geographic data from a study of 2 autochthonous cases of anaplasmosis, Washington, USA, 2022–2023. Blue indicates counties where health investigators have reported *I. pacificus* ticks (*12*); dark blue indicates counties of likely exposure for the 2 cases reported.

Clinicians should be aware of the local presence of *A. phagocytophilum* and the distribution of *I. pacificus* ticks in Washington. *I. pacificus* ticks can also transmit *Borrelia burgdorferi* sensu stricto, the causative agent of Lyme disease, and *Borrelia miyamotoi,* the causative agent of hard tick relapsing fever (*2*). Health officials have increased tick surveillance in Washington, noting increased *I. pacificus* ticks activity in spring and most ticks collected during March−May (*2*,*13*). To support prompt diagnosis and treatment of suspected tickborne disease among Washington residents, clinicians should consider travel history, exposure to tick habitats, seasonality in tick activity, and the patient’s clinical manifestations. More robust tick surveillance in the state could help better define vector distribution, abundance, and infection risk to inform public health prevention messaging and provider education.
